# Population genetics of *Plasmodium vivax* with transmission decline and rebound in two endemic areas of Papua New Guinea

**DOI:** 10.3389/fgene.2025.1621920

**Published:** 2026-02-03

**Authors:** Abebe A. Fola, Somya Mehra, Zahra Razook, Dulcie Lautu-Gumal, Elma Nate, Stuart Lee, Johanna Helena Kattenberg, Cristian Koepfli, James Kazura, Maria Ome-Kaius, Moses Laman, Leanne J. Robinson, Ivo Mueller, Alyssa E. Barry

**Affiliations:** 1 Population Health and Immunity Division, Walter and Eliza Hall Institute, Parkville, VIC, Australia; 2 Department of Medical Biology, University of Melbourne, Carlton, VIC, Australia; 3 IMPACT/School of Medicine, Deakin University, Geelong, VIC, Australia; 4 Life Sciences Discipline, Burnet Institute, Melbourne, VIC, Australia; 5 Vector Borne Diseases Unit, Papua New Guinea Institute of Medical Research, Yagaum, Madang, Papua New Guinea; 6 Centre for Global Health and Diseases, Case Western Reserve University, Cleveland, OH, United States; 7 Central Clinical School, Monash University, Melbourne, VIC, Australia; 8 Department of Parasites and Vectors, Institut Pasteur Paris, Paris, France

**Keywords:** malaria, *Plasmodium vivax*, population genetics, malaria control, diversity, resurgence, identity by descent (IBD)

## Abstract

**Background:**

Global efforts to control and eventually eliminate malaria have been less effective for *Plasmodium vivax* relative to *Plasmodium falciparum* due to its unique biology, including dormant liver stages that cause later relapse, and earlier commitment to transmission stages. After the nationwide distribution of long-lasting insecticide treated nets (LLIN) in Papua New Guinea (PNG), *P. vivax* initially reduced to low prevalence, but again resurged to levels similar to those before LLIN distributions.

**Method:**

To explore changes in *P. vivax* population structure and identify sources of resurgence over this period, we applied a previously validated genome-wide SNP barcode to genotype 336 *P. vivax* isolates obtained from serial cross-sectional surveys conducted over a decade in East Sepik (2005, 2012, 2016) and Madang Province (2006, 2010, 2014).

**Results:**

Population genetic analyses of the resulting parasite genotypes revealed contrasting spatiotemporal patterns between the two provinces. In Madang, the complexity of infection, genetic diversity, and population structure varied with prevalence, with a possible population bottleneck and early clonal expansion at low transmission, and rapid recovery of the population with resurgence. In East Sepik, there was a less dramatic impact on the parasite population after prevalence decline, and ongoing transmission of multiple residual lineages throughout the study period. *P. vivax* decline was also accompanied by an increase in genetic differentiation between the two areas, which reduced with resurgence suggesting changes in parasite migration between areas associated with prevalence.

**Conclusion:**

The earlier implementation of LLIN in East Sepik, smaller rebound, heterogeneity in transmission and relative isolation, compared to Madang may have contributed to these differing patterns. The results demonstrate that long term sustained control efforts are essential to make a lasting impact on the *P. vivax* population, and that SNP barcodes can provide valuable insights into parasite transmission dynamics as a result of control efforts.

## Background

In the past two decades, there has been a dramatic reduction in malaria morbidity and mortality globally using currently available tools such as long-lasting insecticide treated bed nets (LLIN), rapid diagnostic testing (RDTs) and highly effective artemisinin combination therapies (ACTs) (WHO, 2024). This achievement has led many endemic countries to work towards malaria elimination by 2030 (Cibulskis et al., 2016; Rabinovich et al., 2017). However, the emergence of artemisinin resistance (Ippolito et al., 2021), high transmission heterogeneity at local scales (Omedo et al., 2017; Kattenberg et al., 2020a; Gul et al., 2021; Fadilah et al., 2022) and importation of cases from higher transmission regions through human mobility (Chang et al., 2019; Buyon et al., 2020; Neafsey et al., 2021) threatens these elimination plans. Furthermore, in areas where both major human malaria parasites, *Plasmodium vivax* and *Plasmodium falciparum* co-circulate, *P. vivax* is more resilient to control efforts, owing to its ability to relapse, cause lower density infections, and transmit prior to clinical symptoms (Adams and Mueller, 2017; Ferreira and de Oliveira, 2015).

For malaria endemic countries to drive further reductions in transmission and progress towards elimination, new and more efficient surveillance approaches are needed. As infections become rarer and more clustered in space and time, traditional estimates of malaria transmission such as the entomological inoculation rate and infection prevalence, become more difficult and expensive to measure (Tusting et al., 2014). Genomic surveillance applies novel and sensitive molecular tools to parasite isolates collected through community surveys or clinical cases to address a number of malaria control “use cases” (Dalmat et al., 2019), including monitoring changing transmission dynamics (Auburn and Barry, 2017; Neafsey et al., 2021), to track the emergence and spread of new strains such as drug and diagnostic resistant strains (Miotto et al., 2020; Fola et al., 2023), and to monitor population connectivity and the risk of imported infections (Taylor et al., 2017). Population genomic surveillance can reveal whether the transmission is endemic (heterogeneous), epidemic (clonal expansion) or a chain (no recombination) (Colijn and Gardy, 2014; Fola et al., 2018). These genomic analyses provide insights into the transmission dynamics underlying prevalence estimates, and help to determine whether control programs have interrupted endemic transmission, to identify the factors sustaining ongoing or resurgent transmission, and whether existing efforts are adequate to eliminate malaria (Volkman et al., 2012; Barry et al., 2015).

Papua New Guinea (PNG) has the highest *P. vivax* transmission in the world (Cleary et al., 2022). Intensified nationwide malaria control activities since 2004, with nationwide implementation of LLIN by 2008, significantly reduced malaria transmission in PNG between 2008 and 2010 (Hetzel et al., 2015). However, since 2014, malaria has rebounded substantially in the north coast (Koepfli et al., 2017; Kattenberg et al., 2020a) and throughout the country (Hetzel et al., 2017). Earlier studies on *P. vivax* population genetics before malaria control was intensified i.e. pre-LLIN (2005-6) revealed high genetic diversity and a lack of population structure (Koepfli et al., 2013; Jennison et al., 2015). We also investigated the spatiotemporal variation of these *P. vivax* populations from 2005 to 2016 using ten highly polymorphic microsatellite markers, finding that despite a significant decline in parasite prevalence, there was no change in population structure and parasite populations maintained high genetic diversity (Kattenberg et al., 2020b). Nevertheless, some signals of transmission disruption were observed, such as an increase in genetic differentiation (*F*_ST_) between provinces, and the emergence of significant multilocus linkage disequilibrium (*LD*) and focal clusters of phylogenetically related parasites (Kattenberg et al., 2020b).

Microsatellites are fast evolving with high allelic diversity per locus (Li et al., 2002) and previous studies typed less than one marker per chromosome and therefore will have a limited ability to accurately measure relatedness and detect more distant relationships amongst parasite isolates. We have developed an informative single nucleotide polymorphism (SNP) barcode with 176 SNPs spanning all *P. vivax* chromosomes, and demonstrated its improved performance and higher resolution to detect variability in diversity and geographic population structure compared to microsatellites using the 2005-6 baseline samples, collected pre-LLIN (Fola et al., 2020). We hypothesized that the small number of microsatellite markers, their high polymorphism and possibility of technical artifacts (Sutton, 2013; Ruybal-Pesántez et al., 2024), may limit the accurate detection of parasite population structure and Identity by Descent (IBD) as a measure of parasite relatedness. Therefore, we aimed to assess *P. vivax* population genetic signals associated with changing transmission using the SNP barcode (Fola et al., 2020). Using a subset of these SNPs, we measured complexity of infection and proportion of polyclonal infections, population level measures of genetic diversity and structure, and parasite relatedness. The results provide unique and unexpected insights into the spatio-temporal dynamics of *P. vivax* populations associated with declining transmission and rebound, highlighting the importance of marker panel choice and population genetics in characterising transmission dynamics, to monitor *P. vivax* populations.

## Results

### Sample genotyping

**FIGURE 1:**
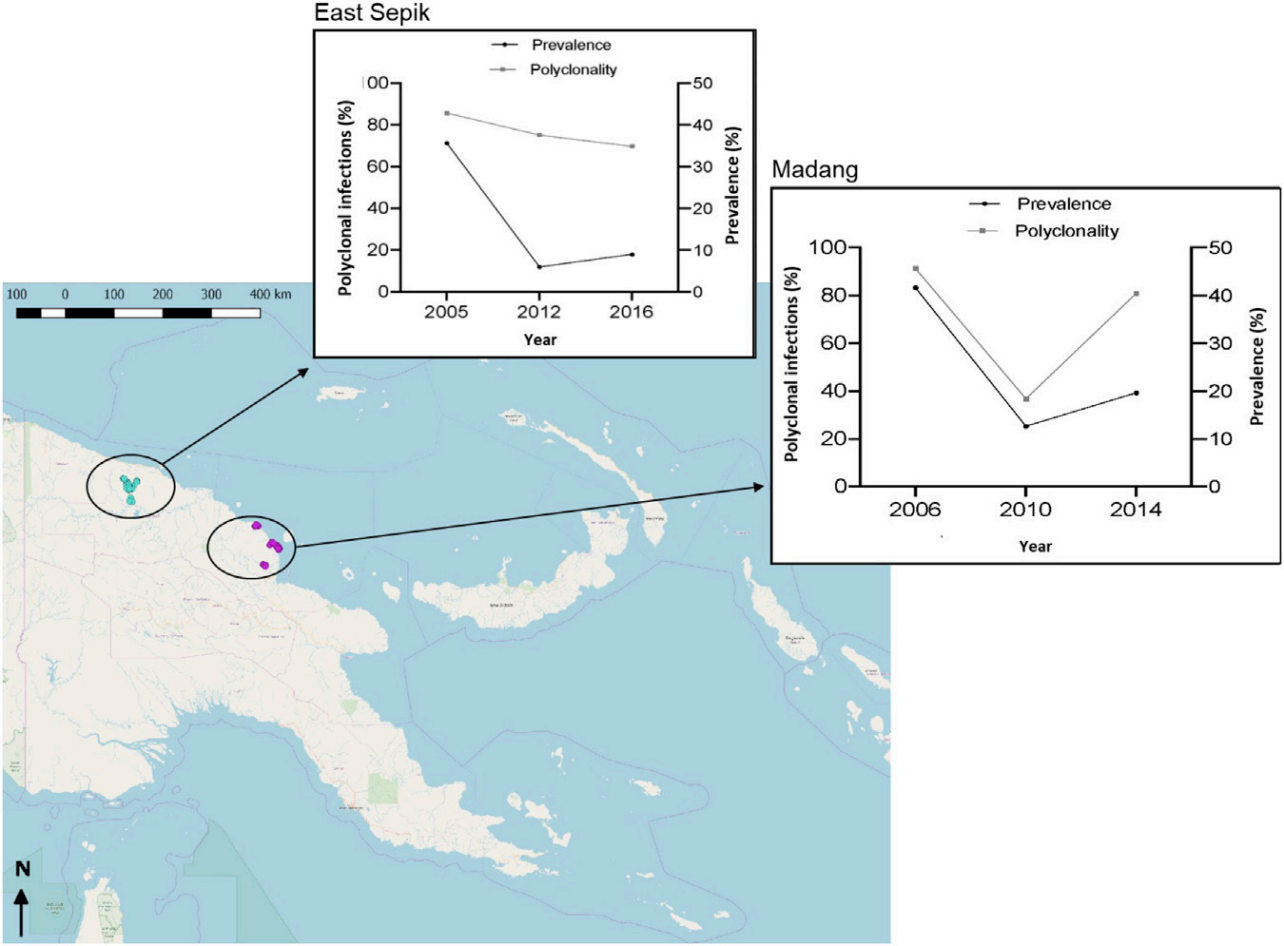
Map of the study area and *P. vivax* prevalence and polyclonal infections trends from 2005 to 2016. The coloured dots in the map indicate villages included in serial cross-sectional surveys in each Province. Blue, East Sepik villages; Pink, Madang villages. The inset graphs associated with each Province shows *P. vivax* malaria prevalence trend measured by qPCR and proportion of isolates that were polyclonal i.e. COI greater than one according to the Real McCoil analysis of SNP genotypes (polyclonality %, Table 1).

One hundred (100) SNPs from a previously validated 176 SNP barcode (Fola et al., 2020), Supplementary Table S1) were used to genotype *P. vivax* isolates from East Sepik (ESP) and Madang Provinces of PNG (Figure 1). Samples were derived from cross-sectional surveys conducted between 2005 and 2016 in several villages of ESP surrounding Maprik town, and from the Mugil, Malala and Utu catchments of Madang Province (Mueller et al., 2009a; Senn et al., 2012; Koepfli et al., 2017; Kattenberg et al., 2020a). From a total of 376 *P. vivax* qPCR positive samples, 336 (89.3%) were successfully genotyped with at least 70% of the target SNPs called per genotype (see Materials and Methods, Table 1). Among the genotypes, 12 out of the 100 SNPs were excluded because at least 30% of samples failed. The remaining 88 SNPs were used to construct infection haplotypes for population genetic analysis (Supplementary Table 1). More samples were available from Madang due to the larger geographic area covered and three defined catchment areas.

**TABLE 1:** Summary of genotyped *P. vivax* samples and complexity of infection.

Province	Year	N	n	*P.vivax* prevalence (%)	Mean COI	Polyclonality (%)	Reference
East Sepik	2005	47	42	36	1.85	85.7	Mueller et al. (2009b)
East Sepik	2012	23	20	6	1.69	75.2	Kattenberg et al. (2020a)
East Sepik	2016	47	43	9	1.61	69.7	Unpublished
Madang	2006	94	81	42	1.93	91.3	Senn et al. (2012)
Madang	2010	94	87	13	1.36	36.8	Koepfli et al. (2017)
Madang	2014	71	63	20	1.92	80.9	Koepfli et al. (2017)

N = total samples genotyped, n = successfully genotyped at greater than 88 SNPs, COI = complexity of infection as measured by The Real McCOIL using SNP barcode data (Chang et al., 2017). References for prevalence data are provided.

### Complexity of infection and genetic diversity

All samples were screened prior to SNP genotyping for multiplicity of infection using capillary electrophoresis of highly polymorphic microsatellites *msp1f3* and *ms16* (Koepfli et al., 2011; Arnott et al., 2013). Only samples with one or two clones were selected, however 238 of the 336 samples (70.83%) were found to have heterozygous SNPs, including 121 of the originally classified single infections, showing that SNP barcoding with deep amplicon sequencing is more sensitive to detect multiple clones. The remaining 98 samples were confirmed as single infections. Complexity of Infection (COI) was then measured using the SNP data (Table 1).

There was significant temporal variation in mean COI and the proportion of polyclonal infections (polyclonality) using SNPs in both study areas (ESP: p < 0.01; Madang: p < 0.001, Mann-Whitney U test). However, in Madang, there was a large decline in mean COI and polyclonal infections from 2006 to 2010 then an increase in 2014, whereas in ESP these values where more stable showing a subtle decline from 2005 to 2012 and continuing to decrease even despite the increase in prevalence in 2016 Table 1; Figure 1).

**FIGURE 2:**
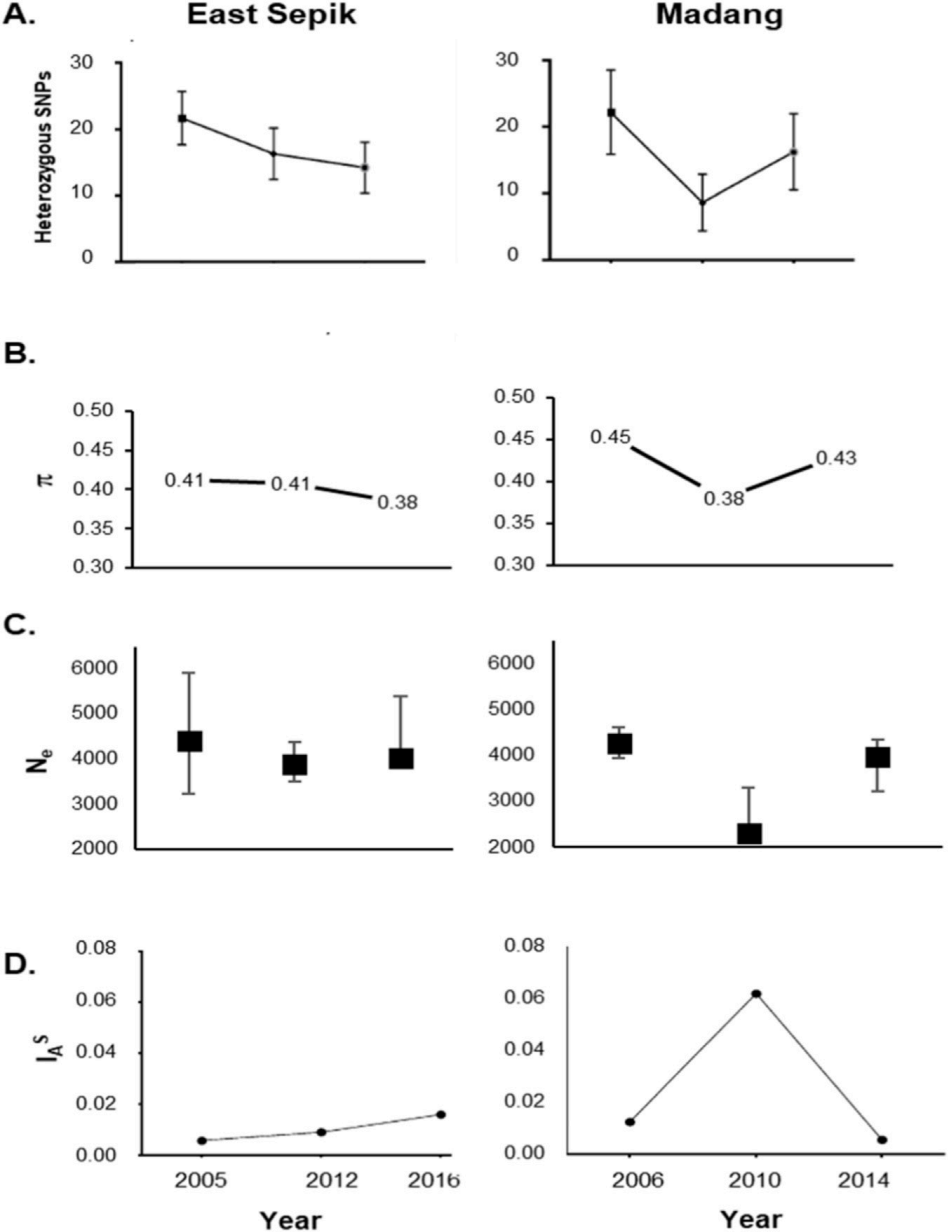
Spatiotemporal changes in genetic diversity of *P. vivax* populations of Papua New Guinea **(A)** Mean number of heterozygous SNP loci, **(B)** Nucleotide diversity (SNP π) **(C)** Effective population size (N_e_). Error bars represent 95% CIs. **(D)** Multilocus Linkage disequilibrium (LD) as measured by the Standardized index of association (I_A_^S^).

As a measure of within-infection genetic diversity, the mean number of heterozygous SNP calls per population was calculated. There was a significant change in this metric with declining transmission in ESP (2005: 21.27, 2012: 16.70, p < 0.01, Mann-Whitney U test) but a much greater decrease for Madang (2006: 22.20, 2010: 8.60), p < 0.001, Mann-Whitney U test) (Figure 2A). In ESP, heterozygous SNPs continued to decline throughout the study period even after rebound in 2016 (14.23). In Madang, the decline of heterozygous SNPs in 2010, was followed by a substantial increase with rebound in 2014 (16.20, Figure 2A). Spatial variation in these values, i.e. comparing ESP to Madang, was only observed at the lowest prevalence time points (ESP 2012, Madang 2010) due to the high proportions of heterozygous SNPs maintained in ESP.

Minor allele frequency (MAF) distributions demonstrate that at baseline the 88 barcode SNPs represented a wide range of allele frequencies (Supplementary Figure S1). With transmission decline, some SNPs shifted from the low allele frequencies to the mid-high frequencies which is consistent with a population bottleneck. This pattern was more pronounced in Madang 2010 and remained through to the third time point, whereas for ESP, the changes were more subtle and SNPs with low allele frequencies increased in the third time point, consistent with population expansion.

The nucleotide diversity statistic, (SNP π), measures genetic diversity among infections. SNP π was high in both Madang (0.45) and ESP (0.41) at baseline (Figure 2B). There were minimal changes in this parameter in ESP as transmission declined from 2005 (0.41) to 2012 (0.41) and upon rebound in 2016 (0.38, Figure 2B). Whereas, in Madang there was a significant reduction in SNP π from 2006 (0.45) to 2010 (0.38, p = 0.024, Mann-Whitney U test) and an increase with transmission rebound in 2014 (0.43, p = 0.062). The change in effective population size (N_e_) over time was also greater in Madang than ESP (ESP 2005: 4,410, 2012: 3,886, 2016: 4,022; Madang 2006: 4,262, 2010: 2,289, 2014: 3,980, Figure 2C).

Low levels of multilocus linkage disequilibrium (LD) in both provinces at baseline are consistent with high levels of transmission and outcrossing (Anderson et al., 2000). LD increased with declining transmission, with a significant change in Madang from 2006 to 2010 (Figure 2D, p < 0.001). Whereas, there was a significant decrease after transmission rebound in Madang 2014 relative to the 2010 timepoint (0.005, p-value <0.01).

### Spatiotemporal changes in *P. vivax* population structure

Low levels of genetic differentiation (*F*_ST_ = 0.075, Supplementary Figure S2) and overlapping genotypes in the Principle Components Analysis (PCA) were observed between provinces at baseline (Supplementary Figure S3A) and are consistent with microsatellite data (Koepfli et al., 2013; Jennison et al., 2015). Comparing the low transmission time points ESP 2012 and Madang 2010, there was a very high degree of spatial genetic differentiation (*F*_ST =_ 0.252, Supplementary Figure S2) and clustering of genotypes (Supplementary Figure S3B). After transmission rebound, the genetic differentiation between the two provinces decreased (*F*_ST_ = 0.182, Supplementary Figure S2) and genotypes showed more overlap in the PCA (Supplementary Figure S3C) indicating an increase in gene flow between provinces. Within ESP there was little genetic differentiation between samples collected in consecutive surveys (2005/2012: *F*_ST_ = 0.07, 2012/2016: *F*_ST_ = 0.08) but high genetic differentiation comparing first and last time points (2005/2016: *F*_ST_ = 0.15, Supplementary Figure S2). However, there was high genetic differentiation among Madang populations between all paired time points (2006/2010: *F*_ST_ = 0.16, 2010/2014 *F*_ST_ = 0.17, 2006/2014 *F*_ST_ = 0.18, Supplementary Figure S2).

**FIGURE 3:**
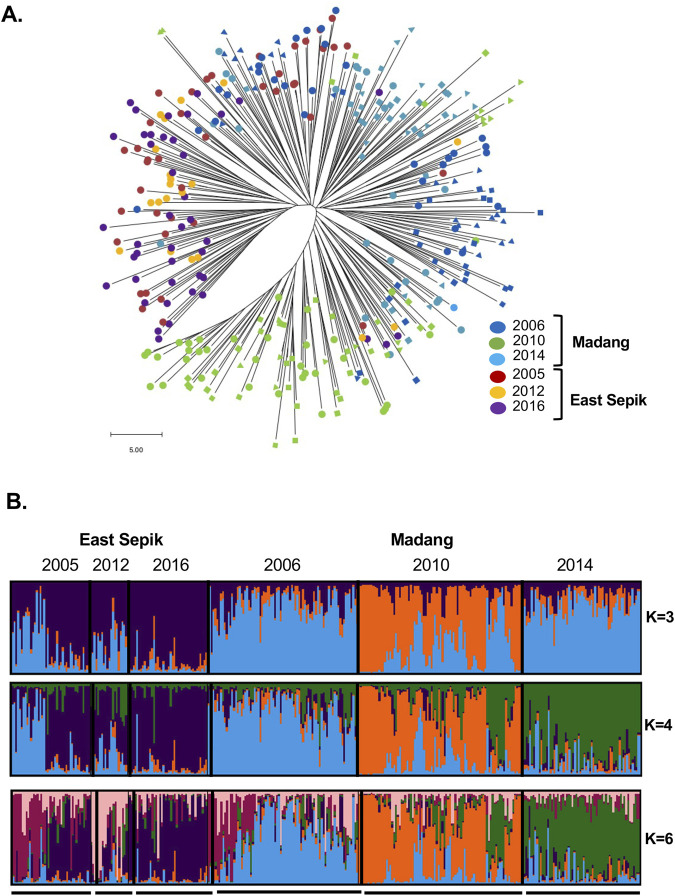
Spatiotemporal changes in population structure of *P. vivax* in Papua New Guinea **(A)** Phylogenetic tree, An unrooted neighbour-joining tree illustrates the genetic relatedness between 336 genotypes of 88 SNPs based on pairwise distance matrices, using the number of differences method, with branch lengths scaled accordingly (scale indicated). Colours indicate geographic area and year, while shapes for Madang, indicate the three catchment areas: circle = Malala, square = Mugil, triangle = Utu. **(B)** Bayesian cluster analysis. Individual ancestry coefficients are shown for K = 3, 4 and 6, each vertical bar represents an individual haplotype and the membership coefficient (Q) within each of the genetic populations, as defined by the different colours.

Phylogenetic analysis confirmed the spatiotemporal population structure and genotype clustering in Madang, with increasing structure in the tree for post-LLIN populations both within and between time points (2010,2014), whereas there was no substantial structure amongst pre-LLIN (2006) Madang genotypes and all ESP genotypes (Figure 3A; Supplementary Figure S4). Small clades of pre-LLIN Madang genotypes (2006) also cluster with ESP genotypes (2005) confirming significant connectivity between provinces at that time. Whereas there was limited clustering of genotypes from the two provinces at the low transmission mid-point (2010/12) consistent with a reduction in gene flow and higher LD. At low transmission, Madang 2010 genotypes comprised two major clades (Figure 3A; Supplementary Figure S4A). The less frequent Madang 2010 clade containing only genotypes from one catchment area (Utu), clustered with the majority of haplotypes from Madang 2014, indicating this lineage was a major source of the Madang resurgence.

Further analysis using STRUCTURE software (Kaeuffer et al., 2007) at K = 3 showed one cluster predominant in ESP 2005 and Madang 2006 and 2014. The other two clusters were predominant in ESP 2012 and 2016, and Madang 2010 populations (Figure 3B). At K = 4, Madang genotypes formed three distinct sub-populations according to the year of collection (2006, 2010 and 2014). Whereas, ESP isolates form two genetic clusters with high proportions of ancestry shared between baseline (2005) and post-control (2012, 2016) populations. Approximately half of the ESP 2005 samples share ancestry with Madang 2006 genotypes. Additional sub-structure was detected at K = 6 for ESP at the village level with genotypes from Sengo clustering in 2005, Sunuhu in 2012 (which was also observed using microsatellites, Kattenberg et al., 2020b), and for Madang in Malala-Amiten village. At K = 4 and 6, a minor Madang 2010 cluster (dark green) accounts for the majority of the Madang 2014 population, consistent with the phylogenetic analysis. The phylogenetic tree also shows genotypes from 2006 in this clade suggesting it may have originated from a persistent lineage circulating throughout the study period (Figure 3A).

### Clonal expansion in Madang at low transmission and ongoing endemic transmission in East Sepik

**FIGURE 4:**
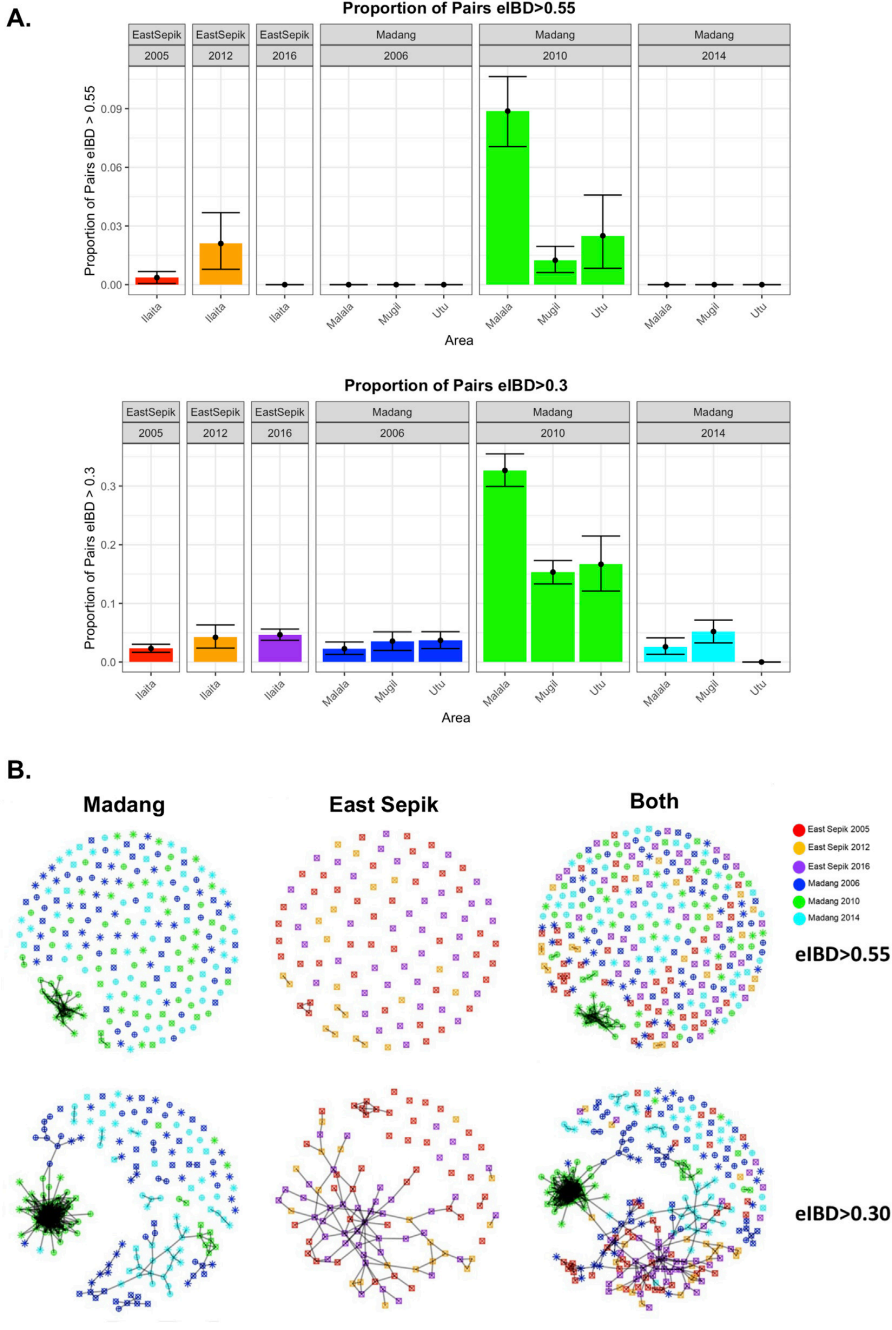
Spatio-temporal changes in genotype relatedness within *P. vivax* populations of PNG. **(A)** Pairwise expected Identity by Descent (eIBD) was calculated using IsoRelate and stratified by geographic location and year **(A)** eIBD > 0.55 and eIBD > 0.30 proportions are shown for each of the populations and time points, with the three catchments for Madang shown separately. **(B)** Spatio-temporal changes in relatedness of *P. vivax* genotypes from Papua New Guinea. Pairwise expected Identity by Descent (eIBD) was calculated using IsoRelate and networks drawn on the basis of relatedness thresholds of 0.55 and 0.30. The colours indicate the study year and location, as shown in the key. Different symbols for Madang represent the different catchments.

To further explore the spatiotemporal distribution of parasite lineages, we measured the expected Identity by Descent (eIBD) parameter and investigated relatedness networks using the “isoRelate” R package (Henden et al., 2018; Taylor et al., 2019). We first evaluated the proportion of genotype pairs with eIBD greater than 0.55 that are likely to be closely related (siblings) or clones, and pairs with at least moderate eIBD using a cutoff of 0.30 (Figure 4A). There was a higher proportion of closely related genotypes observed in the low transmission midpoints for both populations. However, this was more pronounced in Madang where almost 10% of pairs shared high IBD and 30% with moderate levels (Figure 4A). Whereas, in ESP, there was only a small change in the proportions of related pairs. After resurgence, in Madang the proportion of moderate eIBD pairs reduced substantially though remained elevated relative to baseline levels, whereas in ESP eIBD pairs remained elevated relative to baseline.

In the network analysis, most populations show small clusters of related parasites, but Madang 2010 genotypes mostly clustered into two distinct lineages (Figure 4B). Setting the threshold to illustrate moderate relatedness (eIBD greater than 0.30), genotypes from all years connected to the smaller Madang 2010 lineage, aligning with the STRUCTURE and phylogenetic analysis. In ESP, very few closely related pairs were detected, but there was a large cluster of moderately related individuals connecting all three time points. When the two locations were analysed together, related pairs within each site are connected into one large cluster, the majority with moderate eIBD values. The smaller Madang 2010 lineage links with several baseline (2006) lineages via a dispersed cluster of 2014 genotypes suggesting the 2010 lineage recombined with rarer (i.e. undetected in the 2010 dataset) residual genotypes upon resurgence.

A minimum spanning network analysis further demonstrates *P. vivax* had a homogeneous diversity pattern at baseline consistent with endemic transmission (Supplementary Figure S5) while post-LLIN, the cluster of related Madang 2010 genotypes showed a star-like configuration indicative of a recent clonal expansion. ESP showed a more homogeneous pattern throughout all time points, suggestive of sustained low levels of recombination amongst distinct genotypes, and connections to the resurgent populations detected in both Madang 2014 and ESP 2016 (Supplementary Figure S5).

## Discussion

*P. vivax* parasites are more diverse than *P. falciparum* therefore more substantial and sustained reductions in transmission may be needed to reduce parasite diversity from pre-control levels (Neafsey et al., 2012). Population genomic changes in *P. falciparum* strongly correlate with transmission intensity where increasing multilocus LD, higher allele sharing, fewer polyclonal infections and lower genetic diversity have been observed as transmission declines (Anderson et al., 2000; Daniels et al., 2013; Nkhoma et al., 2013). For *P. vivax,* signals such as increasing population differentiation and reductions in effective population size with significant inbreeding have been observed in *P. vivax* populations of Thailand (Kittichai et al., 2017) and Solomon Islands (Waltmann et al., 2018) after more than a decade of intensive control interventions. Our previous investigation of *P. vivax* population structure using ten highly polymorphic microsatellite markers, showed a minimal impact on parasite population diversity and structure, but increased multilocus LD with declining transmission, suggesting an increase in related individuals in the dataset (Kattenberg et al., 2020b). Here we confirm that a 88 SNP barcode and deep amplicon sequencing not only provides higher resolution insights, it identifies significant population structure and increasing proportions of related individuals with declining transmission and highly variable changes between the *P. vivax* populations from the two geographic areas.

In Madang, with declining transmission, SNP barcodes showed a decrease in COI, genetic diversity, and effective population size, and an increase in multilocus LD with large clusters of closely related genotype pairs. At low transmission, Madang 2010 genotypes formed at least two distinct lineages. The lower COI, genetic diversity and effective population size, and allele frequency distributions are suggestive of a prior bottleneck, while the LD, STRUCTURE and IBD results suggest an early clonal expansion. Following resurgence (13%–20% prevalence), the parasite genetic parameters returned to near baseline levels indicating population recovery with continuing transmission of at least one of the Madang 2010 lineages, and importation from East Sepik. In contrast, in East Sepik, where *P. vivax* prevalence declined to only 6% in 2010 with high heterogeneity across villages (Kattenberg et al., 2020a), resurging to only 9% by 2016, more subtle changes were observed. All genetic diversity parameters trended in the same direction at resurgence (2012 vs. 2016), as with transmission decline (2005 vs. 2012). While there were increased proportions of related genotype pairs there were no dominant or large clusters of closely related individuals, suggesting transmission of multiple residual lineages, rather than expansion of specific lineages. Between the two populations, the lower prevalence was accompanied by an increase in population structure and higher genetic differentiation (F_ST_) between provinces, which reversed upon resurgence and likely contributed to population recovery. These variable patterns in population genetic changes observed in the two provinces at the low transmission time points may depend on the prevalence and population diversity at the commencement of interventions, the timing of cross-sectional surveys, as well as the local epidemiology, and other socio-demographic factors.

In regions with high transmission, multiple *P. vivax* genotypes within a single patient are common (Juliano et al., 2010; Fola et al., 2017). A reduction in these polyclonal infections as transmission declines reduces the chance of recombination of distinct *Plasmodium* genotypes (outcrossing) inside the mosquito, reducing the genetic diversity of parasite populations (Zhong et al., 2018). For *P. vivax* however, a decline in COI is expected to occur slowly due to the contribution of relapses to blood stage infections (Robinson et al., 2015). COI trended with prevalence in Madang but not in East Sepik showing that the relationship between *P. vivax* transmission intensity and COI is complex. The lack of association between COI and parasite prevalence in East Sepik, could be explained by the heterogeneity of malaria transmission at village level (Karl et al., 2016; Kattenberg et al., 2020a). This is consistent with other studies (Gunawardena et al., 2014; Noviyanti et al., 2015) and our previous observations across different endemic regions in PNG (Juliano et al., 2010; Fola et al., 2017). Other studies have suggested an association between *P. vivax* COI and transmission intensity but only in areas that have had sustained levels of transmission (Waltmann et al., 2018; Koepfli et al., 2018), suggesting the contribution of relapses to the pool of genotypes may remain high in populations with recent reductions in transmission (Mueller et al., 2009b). Thus, for *P. vivax,* COI can be used as a reliable marker of population reduction to measure longer term transmission intensity patterns, but may be less reliable following more recent changes in transmission.

The detection of reduced within-sample genetic diversity, as indicated by proportions of heterozygous SNPs, suggests an increase in relatedness between clones within infections and/or a reduction in the diversity of new infections and the hypnozoite reservoir. Changes in this parameter were more pronounced in Madang relative to East Sepik over the study period. In addition, the emergence of multilocus LD, together with high genetic differentiation and lower genetic diversity indicates local inbreeding (Anderson et al., 2000; Nkhoma et al., 2013). In Madang, the pattern following resurgence indicated increased random mating amongst genetically distinct individuals, while in East Sepik, LD values continued to trend upwards, albeit to a limited extent, consistent with highly focal transmission and inbreeding (Kattenberg et al., 2020a; Kattenberg et al., 2020b).

The loss of rare alleles in Madang after transmission decline, and detection of a large cluster of related genotypes in the Madang population after intensification of control, suggests a strong bottleneck had occurred which may have been accompanied by a reduction in the hypnozoite reservoir, as indicated by dramatically decreased COI and heterozygous SNPs. Accordingly, there was a significant change in population level diversity and effective population size in Madang after control, but limited changes in East Sepik. The lack of significant change in parasite population size after intervention in East Sepik is consistent with other studies where minimal or no change in parasite genetic diversity was observed with declining transmission based on microsatellites (Waltmann et al., 2018; Kittichai et al., 2017) and genome wide SNP markers (Nkhoma et al., 2013).

The spatio-temporal structure of *P. vivax* populations was also highly variable, with major population structure changes over time in Madang, and limited changes in East Sepik. We confirmed the relatively panmictic *P. vivax* population on the north coast of PNG pre-LLIN, as previously described (Jennison et al., 2015). An increase in population structure at the low transmission mid-point post-LLIN, indicates a significant impact of control on parasite migration and consequent gene flow between provinces. However, there was a 1 to 2 year gap between cross-sectional surveys which may have influenced this result. At low transmission, Madang also had a somewhat fragmented parasite population structure at the local level with Utu parasites clustering separately to Malala and Mugil isolates in the phylogeny, STRUCTURE and IBD analyses. One source of resurgence is indicated by the connectivity of the lineage from Utu in Madang 2010, and the broader Madang 2014 population, which also connects to certain Madang 2006 genotypes in the IBD network. Persistence of genotypes across timepoints, or found only in the higher transmission baseline and final timepoints indicates residual parasite lineages could be a major source of resurgence (Daniels et al., 2013). Interestingly, there was an increased prevalence of *P. vivax* dihydrofolate reductase triple and quadruple mutants, in the Madang parasite population in 2010 (Barnadas et al., 2015) suggesting that drug resistance may also play a role (Barnadas et al., 2015). Drug resistance data however is not available for the 2014 *P. vivax* populations so would need further investigation.

The East Sepik genetic diversity at baseline was similar to post-LLIN levels in Madang, whereas there was a slightly lower baseline COI than Madang. ESP *P. vivax* populations may have already been impacted by control pressure, because there were differences in intervention coverage before upscaling control nationally, with earlier distributions of LLIN in East Sepik (Hetzel et al., 2015). Additionally ESP may have had a highly heterogeneous transmission prior to any control intervention. The heterogeneous transmission (Kattenberg et al., 2020a) and small sample size at low transmission (only 20 isolates in the 2012 population from two villages) may have also prevented the detection of clusters and fluctuations in allele frequencies. Topography and connecting roads, uneven sampling, sample size variation and presence of isolated catchment areas within provinces may explain the variable transmission of malaria across PNG (Cleary et al., 2021), and could influence parasite population structure. Variability in parasite population connectivity via human movement for social and other business activities to urban areas (like Madang town) likely contribute to the patterns observed in this study, with the East Sepik study sites being more remote and less populated. Movement of parasites from other endemic areas in PNG, which would be more likely in Madang, and resultant connectivity between parasite populations regionally may also contribute to maintaining a diverse gene pool (Fola et al., 2018; Tessema et al., 2019; Ferreira and de Oliveira, 2015).

The markers used for detecting population genetic signals of transmission dynamics are critically important (Sutton, 2013; Ruybal-Pesántez et al., 2024). The results we have observed with the 88 SNPs are striking relative to our previous observations with ten microsatellite markers where no population structure was observed, with a single panmictic population found across all timepoints and locations (Kattenberg et al., 2020b). The SNP barcode therefore provided more sensitive and meaningful insights into transmission dynamics, as we previously observed using only the baseline samples (Fola et al., 2020). The greater resolution is likely due to the higher density of markers across the genome which facilitates the detection of moderately related genotypes, whereas microsatellites, with less than one marker per chromosome, likely only detects very closely related genotypes (Cabrera-Sosa et al., 2024). This warrants the exploration of sources of resurgence through SNP barcoding of additional isolates from these and other ongoing epidemiological surveys in PNG (Mueller et al., 2022).

Our study has some limitations that may be addressed in future studies. Firstly, the sample size was variable across timepoints and study sites, with some low sample sizes. This was the result of either lower density parasitaemias, the quality of the DNA or limited availability due to low prevalence. While every effort was made to produce similar sample sizes across populations, this may impact the accuracy of statistical outputs, particularly in populations where the sample size is small. While the 88-SNP barcode used for this study provides useful population-level insights, a barcode with more SNPs may provide greater resolution to detect related individuals or inbreeding, and whole genome sequences (WGS) would provide details on a range of features including regions under selection, such as drug resistance, or adaptations to lower transmission timepoints. Currently however, the cost of WGS (approximately USD150-200) compared to SNP barcodes (approximately USD20) is too high to allow large-scale surveillance. In previous studies, relatedness of parasites between geographic locations have been linked to human mobility data which, as mentioned above, may explain some of the differences between provinces (Chang et al., 2019).

Overall, the results demonstrate the variable impact of malaria control on parasite population genetic structure within the same high transmission region, despite similarities in parasite prevalence and reductions due to control, underscoring the critical role of genomic surveillance to detect underlying transmission dynamics that cannot be inferred from prevalence alone. Whilst the nationwide distribution of LLIN and other control measures in PNG made a major impact on the *P. vivax* transmission dynamics in Madang with the initial reduction in prevalence, it was not sustained. Resurgence and expansion of the residual *P. vivax* populations and increasing movement of parasites via the higher prevalence and human movement appears to have been an important contributor to the patterns observed. In East Sepik, prior control efforts and high heterogeneity of transmission may have contributed to the smaller resurgence and more subtle changes in parasite population genetic parameters. This research highlights the power of genomic surveillance to track the impact of malaria control and identify the possible sources of malaria resurgence. These observations can aid in the prioritisation of targeted control and reduce the re-emergence and spread of residual infections. For *P. vivax*, maintaining control pressure for long periods will be essential to exert sustainable impacts on transmission and prevent rapid population recovery.

## Methods and materials

### Samples and study sites

Human blood samples for the study were collected during serial cross-sectional studies conducted in the Mugil, Malala and Utu areas of Madang Province in 2006, 2010 and 2014 and Maprik area of East Sepik Province (ESP) in 2005, 2012–13 and 2016 (Mueller et al., 2009a; Senn et al., 2012; Koepfli et al., 2017; Kattenberg et al., 2020a). These geographic areas are lowland, inland and coastal areas that are hyperendemic for malaria with a high prevalence of both major human malaria parasites *P. falciparum* and *P. vivax*. In Madang, catchments are found approximately 30–50 km from the urban centre of Madang Town, with Utu inland, and Mugil and Malala located along the North Coast Highway, whilst in ESP, all villages were located in an inland area to the south, east and west of Maprik town (Figure 1). In both provinces, there were major reductions in *P. vivax* prevalence from the first time point (2005/6) to the second time point (Madang 2010 and ESP 2012–13) due to intensified control efforts in the intervening years (Figure 1; Table 1). Following this (Madang 2014 and ESP 2016), there was a documented resurgence of malaria overall (Cleary et al., 2022), and an increase in *P. vivax* prevalence in the study sites (Koepfli et al., 2017, unpublished data) (Figure 1; Table 1).

Ethical approval for the study was provided by the Institutional Review Board of the PNG Institute of Medical Research No. 12.21, the Medical Research Advisory Council of PNG No. 13.08 and the Walter and Eliza Hall Human Research Ethics Committee No. 13.14.

### Sample processing and genotyping

Processing of samples included extraction of genomic DNA using Qiagen or Favorgen kits, followed by screening for *Plasmodium* species infection using both light microscopy and species-specific qPCR targeting the 18s rRNA genes (Kattenberg et al., 2020a; Koepfli et al., 2017). In previous studies, *P. vivax* positive samples were subject to genotyping for multiplicity of infection (MOI) using two highly polymorphic microsatellite markers (e.g. *msp1f3, ms16*) (Arnott et al., 2013). Samples with only one or two clones detected based on this genotyping were prioritised to avoid issues associated with reconstructing haplotypes from the SNP barcoding.

To streamline the protocol and reduce costs, a subset of 100 the 176 validated SNPs (Fola et al., 2020) were selected to obtain relatively even coverage of the genome (Supplementary Table S1). A total of 376 *P. vivax* positive isolates were genotyped using these 100 SNP markers, with all procedures conducted as previously described SNPs (Fola et al., 2020) with modifications in the number of pools of multiplexed SNP loci to accompany the smaller number of markers. Following data cleaning and filtering of SNPs and samples due to missing data, a total of 88 SNPs were used for further analysis.

### Bioinformatic analysis

The raw FASTQ files were demultiplexed by binning based on the MID index, the read quality was checked using FastQC (Version 0.8.0) (Andrews and Bittencourt, 2010) and combined FastQC output for all samples were visualized using MultiQC (Ewels et al., 2016). Low-quality reads (<Q30), adaptors, primers, and reads shorter or longer than expected size of amplicon were trimmed using Trimmomatic (Bolger et al., 2014). Only reads that passed stringent quality filters progressed for alignment and variant calling. Unmapped BAM files were generated from quality filtered and trimmed FASTQ files using the FastqtoSam function (http://broadinstitute.github.io/picard/). The combined pipeline was then used to generate indexed, mapped BAM files. This pipeline consists of SamToFastq, bwa-mem and MergeBamAlignment to map reads, and generates a clean and indexed mapped BAM file. In brief, the sequenced reads were mapped to the *P. vivax* Salvador I strain reference genome using BWA MEM (Vasimuddin et al., 2019). Overall quality and genome coverage of mapped bam files were checked using QualiMap v.2.2.1 (García-Alcalde et al., 2012) and detailed bioinformatics described in our previous publication (Fola et al., 2020).

In sequencing large sample sets on multiple MiSeq runs, batch effects are inevitable, thus we used the same sequencing protocol and bioinformatics tools to analyze data from different sequencing runs. However, there were still some batch effects observed in the data set when SNPs were called for each run separately due to allele frequency variation between batches (Supplementary Figure S6A). Combining the data for variant calling was used to minimize the batch effect introduced by the different runs. This helped in reducing induced batch effects as seen in the multidimensional scale plots (Supplementary Figure S6B).

### Complexity of infection and heterozygous SNPs

In malaria endemic countries like PNG, individuals often harbour complex genetically distinct clones of the same species, defined by the detection of multiple alleles in genotyping assays. The number of clones is known as the multiplicity of infection (MOI) and can be measured using the proportion of heterozygous SNP calls (Auburn et al., 2012). Expecting to observe less heterozygous SNP calls with declining transmission, we determined the proportion of heterozygous SNP calls for samples genotyped from different time points. The most likely number of clones present in each sample was calculated using the Real McCOIL (Chang et al., 2017) and only monoclonal or dominant genotypes constructed from the major alleles in polyclonal samples were used for population genetic analysis. Moreover, assuming recent intensive malaria control activities may lead to a change in the frequency of minor alleles with a loss of rare variants with declining transmission, minor allele frequencies (MAF) were determined for all SNPs for all genotyped samples from different time points. The MAF was computed as the proportion of genotyped samples carrying the genotype that was least common.

### Multilocus linkage disequilibrium and effective population size

Multilocus linkage disequilibrium (LD) was determined in each parasite population collected from different time points expecting evidence of inbreeding or increased LD over time with declining transmission. Multilocus LD was measured using the statistic I_A_S (standardized index of association), which compares the observed variance in the numbers of alleles shared between genotypes with that expected when genotypes share no alleles at different loci (Haubold and Hudson, 2000). I_A_^S^ was calculated using LIAN version 3.6 software by applying a Monte Carlo test with 100,000 re-sampling steps. LIAN compares the observed association of markers to the values expected for random association based on the population diversity. To assess the effect of control on parasite effective population size (N_e_), we examined population size using the method implemented in NeEstimator v. 2.0 software (Do et al., 2014), we considered that *P. vivax* has a similar generation time as *P. falciparum* (2 months) (Nkhoma et al., 2013) with a maximum N_e_ value of 20,000 assigned in the software since the *P. vivax* population has a higher population size than *P. falciparum* (Koepfli et al., 2013; Jennison et al., 2015).

### Genetic diversity and population structure

The SNPs were converted to numeric data (i.e., target loci contain reference alleles = “0” and target loci contain no reference alleles = “1”) and used as an input file to determine genetic diversity and population structure analysis. Then the creation of input files for genetic analysis was performed using CONVERT version 1.3.1 (Lischer and Excoffier, 2012). We calculated nucleotide diversity (SNP π) and Wright’s F_ST_ using DnaSP Version 5.0 (Librado and Rozas, 2009). We measured parasite population structure and genetic differentiation between parasites sampled before and after an enhanced intervention. The Bayesian clustering software, STRUCTURE version 2.3.4 (Pritchard et al., 2000; Pritchard et al., 2000) was used to determine the number of population clusters (K) and whether haplotypes clustered according to geographical origin. We performed STRUCTURE runs with a burn-in period of 100,000 followed by 100,000 Markov chains (MCMC iterations). Evanno’s method of ΔK statistics implemented in the STRUCTURE HARVESTER (Earl and vonHoldt, 2012) to determine best genetic clusters. CLUMPAK-Cluster Markov Packager Across K web-based server was used for summation and graphical representation of STRUCTURE results (Kopelman et al., 2015).

### Phylogenetic and relatedness analysis

We calculated genotype relatedness in each year based on the similarity of their SNP barcodes within the population. An increase in genotype relatedness (carrying similar SNP barcodes) is expected since there is a low chance of recombination of different clones inside mosquito with declining transmission. Pairwise comparisons of the number of alleles shared (PS) was computed based on the metric 1-PS using the “Ape” R package ‘dist.gene’ command. This was used to conduct multidimensional scaling (MDS) and principle components analysis (PCA). The phylogenetic relationships were inferred using the Neighbor-Joining method (and computed using the number of differences method in MEGA version 11 (Tamura et al., 2021). The goeBURST algorithm within PHYLOViZ 2.0 (Francisco et al., 2012) was used to generate a minimum spanning tree.

To measure pairwise relatedness values, we calculated identity by descent (IBD) using IsoRelate (Henden et al., 2018). IsoRelate uses a hidden Markov model (HMM) to detect genomic regions that are identical by descent (IBD) and aids simultaneous detection of parasite population clustering (https://github.com/bahlolab/isoRelate). We assume that all isolates are monoclonal and retained only isolates with missingness less than or equal to 30%, and SNP loci with missingness less than or equal to 30% and MAF less than or equal to 0.01, and 1 cM was considered equivalent to 17 kbp as for *P. falciparum*, (Henden et al., 2018). IBD parameters were then measured for each pair of genotypes using the ‘getIBDparameters’ command. Pairwise posterior probabilities of IBD sharing was estimated at each locus (i.e. the probability that isolate A and isolate B are IBD at a particular SNP locus, under the implemented HMM of relatedness and the parameters inferred in step 1) using the ‘getIBDposterior’ command. We then computed the expected fraction IBD (eIBD), defined to be the mean probability of IBD sharing across all loci (Taylor et al., 2017) for each pair of isolates. Note that the isoRelate output was corrected by a factor of 2. Sparse SNP barcode data cannot reliably infer low-level IBD sharing, with previous validation of a 155-SNP barcode for *P. falciparum* against whole genome sequence data indicating a lower resolution limit of 0.25 (i.e. ∼30 SNPs, data not shown). We selected eIBD threshold values of 0.30 and 0.55 for exploration of relatedness within and between *P. vivax* populations.

## Data Availability

The original contributions presented in the study are publicly available. This data can be found in the European Nucleotide Archive (ENA) at EMBL-EBI with the accession number PRJEB105390.

## Supplementary material

Supplementary File 1 Processed and cleaned dataset.

Supplementary Datasheet 1 Figures S1 - S6, Table S1.
