# Correction: Population genetics of *Plasmodium vivax* with transmission decline and rebound in two endemic areas of Papua New Guinea

**DOI:** 10.3389/fgene.2026.1805349

**Published:** 2026-02-16

**Authors:** Abebe A. Fola, Somya Mehra, Zahra Razook, Dulcie Lautu-Gumal, Elma Nate, Stuart Lee, Johanna Helena Kattenberg, Cristian Koepfli, James Kazura, Maria Ome-Kaius, Moses Laman, Leanne J. Robinson, Ivo Mueller, Alyssa E. Barry

**Affiliations:** 1 Population Health and Immunity Division, Walter and Eliza Hall Institute, Parkville, VIC, Australia; 2 Department of Medical Biology, University of Melbourne, Carlton, VIC, Australia; 3 IMPACT/School of Medicine, Deakin University, Geelong, VIC, Australia; 4 Life Sciences Discipline, Burnet Institute, Melbourne, VIC, Australia; 5 Vector Borne Diseases Unit, Papua New Guinea Institute of Medical Research, Yagaum, Madang, Papua New Guinea; 6 Centre for Global Health and Diseases, Case Western Reserve University, Cleveland, OH, United States; 7 Central Clinical School, Monash University, Melbourne, VIC, Australia; 8 Department of Parasites and Vectors, Institut Pasteur Paris, Paris, France

**Keywords:** malaria, *Plasmodium vivax*, population genetics, malaria control, diversity, resurgence, identity by descent (IBD)

The title of this article was erroneously given as: “Population genetics of *Plasmodium vivax* with transmission and rebound in two endemic areas of Papua New Guinea”. The correct title of the article is “Population genetics of *Plasmodium vivax* with transmission decline and rebound in two endemic areas of Papua New Guinea”.

The original article has been updated.

