# Supplementary material for: Population genetics of Plasmodium vivax with transmission decline and rebound in two endemic areas of Papua New Guinea
Source: Front Genet. 2026 Feb 3;16:1621920. doi: 10.3389/fgene.2025.1621920 (PMC12951784; doi:10.3389/fgene.2025.1621920)
Supplement: Supplementary file 2 [file DataSheet1.docx]

**Supplementary Materials**

**Population genetics of Plasmodium vivax with transmission and rebound in two endemic areas of Papua New Guinea**

Abebe A. Fola^1,2,a^, Somya Mehra^1,2^, Zahra Razook^1,4^, Dulcie Lautu-Gumal^1,2,3^, Elma Nate^5^, Stuart Lee^1^, Johanna Helena Kattenberg^1,5,b^, Cristian Koepfli^1,c^, James Kazura^6^, Maria Ome-Kaius^4^, Moses Laman^4^, Leanne J. Robinson^1,2,3,9^, Ivo Mueller^1,2,10^, Alyssa E. Barry^1,2,3,4^*

1. Population Health and Immunity Division, Walter and Eliza Hall Institute, Parkville, Victoria, AUSTRALIA

2. Department of Medical Biology, University of Melbourne, Carlton, Vic, AUSTRALIA

3. Life Sciences Discipline, Burnet Institute, Melbourne, Vic. AUSTRALIA

4. IMPACT/School of Medicine, Deakin University, Geelong, Vic, Australia

5. Papua New Guinea Institute of Medical Research, Madang, Madang Province, PAPUA NEW GUINEA

6. Centre for Global Health and Diseases, Case Western Reserve University, Cleveland, Ohio, USA

6. Central Clinical School, Monash University, Melbourne, Victoria, AUSTRALIA

7. Department of Parasites and Vectors, Institut Pasteur Paris, Paris, FRANCE

a. Current address: Department of Pathology and Lab Medicine, Brown University, Providence, RI, USA

b. Institute of Tropical Medicine, Antwerp, BELGIUM

c. Department of Biological Sciences & Eck Institute for Global Health, University of Notre Dame, Notre Dame, Indiana, USA

*Corresponding Author:

Centre for Innovation in Infectious Disease and Immunology Research (CIIDIR), Health, Education and Research Building, Deakin University, 285-99 Ryrie St., Geelong, Vic., Australia, 3220

p. +613 52273504

e. a.barry@deakin.edu.au

***Figure S1. Temporal changes in minor allele frequency (MAF) in parasite populations of East Sepik and Madang Provinces, Papua New Guinea.***

*Minor allele frequency (MAF) was computed as the proportion of genotyped samples carrying the genotype that was least common. Apart from Mad 2010, there was no significant reduction in low frequency alleles, which indicates a bottleneck.*

***FIGURE S2****.* ***Spatio-temporal changes in genetic differentiation between P. vivax populations of Papua New Guinea.*** *Pairwise matrix showing Wrights Fst values for population pairs.*

***Figure S3. Spatial clustering patterns of P. vivax genotypes in Papua New Guinea at different timepoints.***

*Principal component analysis (PCA) of P. vivax genotypes were done between populations at A) baseline, high transmission (ESP 2005, Mad 2006) B) midpoint, low transmission (ESP 2012, Mad 2010) and C) high transmission, rebound (ESP 2016, Mad 2014). PCA analysis was done using ‘prcomp’ function in R software and plot using interactive plot command ‘plot_ly’. Plots show the three first principal components and each circle with x-y location of point represents a parasite isolate and the colour is assigned according to the year and geographic origin of parasites.*

***Figure S4. Phylogenetic analysis of Plasmodium vivax genotypes from Papua New Guinea.*** *A) Madang and B) East Sepik genotypes were used to construct unrooted neighbour-joining trees using the “MEGA11” package and illustrate the genetic relatedness between genotypes based on pairwise distance matrices (number of differences). Colours indicate geographic area and year, while shapes for Madang, indicate the three catchment areas: circle = Malala, square = Mugil, triangle = Utu.*

***Figure S5. Minimum spanning networks of Plasmodium vivax genotypes from north coast of Papua New Guinea over time.***

*Each coloured circle represents a unique genotype, connecting lines indicate shared alleles among the connected haplotypes. Length is not correlated with the actual genetic distance or mutation rates. The analysis was done using PHYLOViZ software.*

***Figure S6. Multidimensional scaling analysis of P. vivax genotypes before and after adjustment for batch effects***

*Multidimensional scaling (MDS) analysis was done using ‘cmdscale’ function in R software. Dots indicate individual genotypes. Colours indicate study years and geographic clusters as indicated in the key. The genotypes from the same sequencing run (e.g. ESP 2005 and 2016 isolates) were more clustered together before adjusting (A) than after adjusting for sequencing batch effects (B).*

***Table S1. SNP details, primers and combinations for multiplexing***
